# Characterization of High-Ornithine-Producing *Weissella koreensis* DB1 Isolated from Kimchi and Its Application in Rice Bran Fermentation as a Starter Culture

**DOI:** 10.3390/foods9111545

**Published:** 2020-10-26

**Authors:** Mun So Yeong, Moon Song Hee, Chang Hae Choon

**Affiliations:** Kimchi Research Center, Department of Food and Nutrition, Chosun University, 309 Pilmun-daero, Dong-gu, Gwangju 61452, Korea; ohno1757@gmail.com (M.S.Y.); cocoajjoa@hanmail.net (M.S.H.)

**Keywords:** *Weissella koreensis*, rice bran, fermentation, ornithine, functional food

## Abstract

High-ornithine-producing *Weissella koreensis* DB1 were isolated from kimchi. Ornithine is produced from arginine via the intracellular arginine deiminase pathway in microorganisms; thus, high cell growth is important for producing ornithine in large quantities. In this study, excellent *W. koreensis* DB1 growth (A_600_: 5.15–5.39) was achieved in de Man, Rogosa, and Sharpe (MRS) medium supplemented with 1.0–3.0% arginine (pH 5.0) over 24–48 h at 30 °C, and the highest ornithine (15,059.65 mg/L) yield was obtained by culture in MRS containing 3.0% arginine for 48 h. *W. koreensis* DB1 was further investigated as a functional starter culture for rice bran fermentation. After 48 h of fermentation at 30 °C, the fermented rice bran was freeze-dried and ground. The prepared fermented rice bran contained 43,074.13 mg/kg of ornithine and 27,336.37 mg/kg of citrulline, which are used as healthcare supplements due to their beneficial effects. Furthermore, the organoleptic quality of the fermented rice bran was significantly improved, and the fermented product contained viable cells (8.65 log CFU/mL) and abundant dietary fiber. In addition, an investigation of its safety status showed that it has no harmful characteristics. These results indicate that the fermented rice bran product produced is a promising functional food candidate.

## 1. Introduction

l-Ornithine is used as a healthcare supplement, as it is required for proper immune system and liver function. This non-proteinogenic amino acid has been used to treat liver disorders and is a component of the urea cycle, which removes ammonia from the blood [1]. The administration of ornithine has been reported to reduce stress, improve sleep quality, reduce physical fatigue, increase mental ability [2,3,4], improve skin aesthetics, promote the synthesis and production of collagen [5,6], and effectively increase muscle growth and prevent obesity by enhancing basal metabolism [6]. Ornithine is widely distributed in various foods, especially in mushrooms and *Corbicula japonica*, which contain 10–170 mg and 160 mg per 100 g, respectively [1,7]. However, these amounts are substantially lower than those required to satisfy daily requirements (400–1000 mg), and the same applies to fish and meat consumption [8].

Lactic acid bacteria (LAB) are industrially important microorganisms, as they are used in starter cultures for fermented food processing because they improve the sensory qualities and food preservation. Moreover, LAB are now the subjects of intensive research because of their health benefits [9]. Kimchi is a LAB-rich food and, thus, a good source of potentially beneficial LAB. Actually, during our previous studies, we isolated LAB from kimchi and investigated their abilities to produce bacteriocin, antifungal compounds, exopolysaccharide, mannitol, and γ-aminobutyric acid and their cholesterol-lowering effects and acid and bile tolerances in the contexts of the food and feed industries [9,10,11,12,13,14].

Arginine is the precursor of ornithine and is converted by the arginine deiminase (ADI) pathway to l-arginine, l-citrulline, and NH_3_; thereafter, the l-citrulline produced is converted by ornithine transcarbamoylase to l-ornithine and carbamoyl phosphate. The latter is converted to NH_3_, CO_2_, and ATP by carbamate kinase [15]. The ADI pathway is widely utilized in bacteria, including LAB. Arginine is readily detected in kimchi, grape juice, and wine and is readily degraded by some LAB [16,17,18]. However, interestingly, in kimchi, the arginine contents fall to almost undetectable levels during fermentation, whereas the total amino acid contents increase slightly due to the degradation of proteinaceous components [17,19], which suggests that the microorganisms possessing the ADI pathway are involved in the process. Ornithine production by the LAB strains *Lactobacillus brevis*, *Weissella confusa*, and *Weissella koreeensis* isolated from kimchi has been previously investigated, but the amounts of ornithine produced were below 1.5 g/L [6,17].

In this study, we isolated and identified an effective ornithine-producing LAB from kimchi. The isolates were then assessed for safety, which included investigations of their hemolytic activity, undesirable biochemical characteristics, and antibiotic resistance, and this was followed by an investigation on the effects of culture conditions on the growth of the isolates and their ability to produce ornithine. To explore practical applications, we selected one isolate as a functional starter culture for rice bran fermentation and characterized the fermented rice bran obtained along with a sensory evaluation.

## 2. Materials and Methods

### 2.1. Kimchi Sampling and Isolation of LAB

A total of 38 kimchi samples were collected from 29 different regions in Korea, macerated using a hand blender (Hanil, Seoul, Korea), and filtered through a thin sterile cloth. The filtrates were serially diluted and spread on de Man, Rogosa, and Sharpe (MRS; Difco, Sparks, MD, USA) agar containing 2.0% CaCO_3_. The plates were incubated at 30 °C for 48 h; thereafter, colonies that formed a clear zone were selected as tentative LAB strains.

### 2.2. Identification of Isolates

Isolates were identified based on their morphological properties under a microscope, by a catalase test, by Gram-staining, and according to 16S rDNA sequence analysis [20], which was compared with sequences available in GenBank (https://www.ncbi.nlm.nih.gov/) using CLUSTAL W (https://www.genome.jp/tools-bin/clustalw).

Random amplified polymorphic DNA (RAPD)-PCR was performed using the M13 primer, which is specific for *Weissella* species [21]. The PCRs were performed in a PCR unit (CP2-03 Thermal Cycler, Corbett Research, Mortlake, Australia) using annealing temperatures of 30, 35, 40, 45, 50, or 55 °C. The PCR products were separated by electrophoresis on 1.5% (*w*/*v*) agarose-TAE (40 mM tris-acetate, 1 M EDTA, pH 8.2) gels using the BioFACT™ 1 Kb Plus DNA Ladder (Biofact, Daejeon, Korea) as a size marker [13].

### 2.3. Thin Layer Chromatography (TLC) and High-Performance Liquid Chromatography (HPLC)

To prepare filter-sterilized culture supernatants, isolates were cultivated in MRS + 1.0% arginine broth for 48 h at 30 °C, centrifuged (9950× *g*, 15 min, 4 °C), and then filtered (0.4 μm pore size, Advantec, Dublin, CA, USA). Ornithine production by isolates was determined qualitatively by spotting filter-sterilized culture supernatants onto TLC silica gel 60 F_254_ plates (Merck, Darmstadt, Germany). The mobile phase used was a 1-butanol/acetic acid/distilled water (12:3:5) mixture, and the plates were subsequently stained with a 0.5% (*w*/*v*) ninhydrin solution [22,23].

HPLC was used to determine the amino acid contents in the culture supernatants of the isolates and fermented rice bran. For HPLC analysis, the culture supernatants were derivatized using O-phthalaldehyde–fluorenylmethyl chloroformate (OPA-FMOC) [24] and analyzed using an HPLC unit (Ultimate 3000) equipped with a Fluorescence detector, an UV detector (Agilent 1260, Agilent, Santa Clara, CA, USA), and an Inno C18 column (5 μm, 4.6 × 150 mm; Youngjin, Seongnam, Korea). Elution was performed using mixtures of Solution A (40 mM sodium phosphate, pH 7.0) and Solution B (methanol/acetonitrile/water = 45:45:10) using the following program: A/B at 95:5 for 0–24 min, 45:55 for 24–25 min, 10:90 for 25–34.5 min, and 95:5 after 34.5 min at a flow rate of 1.5 mL/min [25].

### 2.4. Effects of Culture Conditions on Growth

#### 2.4.1. Temperature

Overnight-cultured LAB isolates were used to inoculate (1.0%, *v*/*v*) MRS broth and then incubated at 5, 10, 15, 25, or 30 °C for 24–288 h. Growth was measured every 4 or 8 h by measuring absorbance at 600 nm (A_600_; Ultrospec 2100 Pro, Biochrom, Cambridge, UK). In addition, viable counts at maximum A_600_ were determined using the plate method [26].

#### 2.4.2. pH

Overnight-cultured LAB isolates were used to inoculate (1.0%) MRS and MRS + 1.0% arginine broth; the pH values were adjusted to pH 5.0, 6.0, 7.0, or 8.0, and the mixtures were incubated at 30 °C for 24 h. Thereafter, cell growth was determined by measuring absorbance at 600 nm (Biochrom, Cambridge, UK).

#### 2.4.3. Arginine (Precursor) Concentrations

The dependence of LAB growth on the arginine (as a precursor of ornithine) concentration in the MRS broth was examined. Arginine was added to MRS broth at 0.5, 1.0, 2.0, or 3.0% (*w*/*v*), and the pH values were adjusted to optimum values based on the results described in Section 2.4.2 above. Cultures supplemented with arginine at 0.5–3.0% without pH adjustment were used as controls. Incubation was performed for 24–48 h at 30 °C, and cell growth was determined at 600 nm (Biochrom, Cambridge, UK)). The conversion of arginine to ornithine was assessed by TLC.

### 2.5. Safety Assessment

#### 2.5.1. Harmful Enzyme Activities

Enzymatic activities were assayed using an API-ZYM kit (BioMérieux, Lyon, France) according to the manufacturer’s instructions. Harvested LAB cultures were resuspended in sterile distilled water (McFarland standard 5), spotted (65 μL) into each cupule, and incubated at 37 °C for 4 h. ZYM-A and ZYM-B kit reagents were then added to each cupule, and enzyme activities were determined after allowing the reactions to continue for 5 min.

#### 2.5.2. Antibiotic Susceptibility and Hemolysis

LAB were evaluated for their susceptibilities to antibiotics according to the technical guidelines issued by the European Food Safety Authority (EFSA) [27]. LAB strains cultivated overnight in MRS broth were harvested (9950× *g*, 15 min, 4 °C) and resuspended in Mueller–Hilton (MH; Difco, Sparks, MD, USA) broth containing 0.5% dextrose at a concentration of ~7.0 log CFU/mL. The minimal inhibitory concentrations (MICs) of ampicillin, chloramphenicol, erythromycin, gentamycin, kanamycin, streptomycin, tetracycline, and vancomycin (Sigma, St. Louis, MO, USA) were then determined. Briefly, each antibiotic was added to aliquots of the MH suspension and incubated for 24–48 h at 30 °C without shaking. Cell growth was assessed by measuring absorbance at 600 nm (Biochrom, Cambridge, UK). Cultures in MH broth containing 0.5% dextrose for 24–48 h at 30 °C in the absence of any antibiotic were used as controls [10].

For the hemolysis test, LAB cells were streaked on blood agar containing 7.0% horse blood (Oxoid, Hampshire, UK). Plates were incubated for 24–48 h at 30 °C to detect α-hemolysis or for 24–48 h at 30 °C and for 24 h at 4 °C to detect β-hemolysis [10,28] and then assessed for the presence of clear zones around colonies. *Bacillus cereus* ATCC 14579 was used as a control for the hemolysis assay [10].

#### 2.5.3. Production of Biogenic Amines

LAB were cultured in MRS + 1.0% arginine for 48 h at 30 °C, harvested (9950× *g*, 15 min, 4 °C), filtered (0.4 μm, Advantec), freeze-dried, and concentrated 5-fold in distilled water. 2 mL of samples were then added to 10 mL of 0.4 M perchloric acid–5.0% trichloroacetic acid, and then 0.2 mg of 1,7-diaminoheptane (internal standard) was added. The mixtures were then homogenized, benzoylated, dissolved in 0.5 mL of 50% methanol, and subjected (20 μL) to HPLC analysis [29]. The HPLC unit was equipped with a SunFire C18 column (3.5 μm, 4.6 × 150 mm; Waters, Boston, MA, USA), a Waters 2996 photodiode array detector at 254 nm, and the Waters Empower software (Waters 2695, Waters, Boston, MA, USA). Gradient elution was performed using aqueous methanol mixes, as follows: 50% methanol for the first 15 min followed by a linear increase to 90% methanol for 10 min and then a decrease to 50% methanol for 5 min, and then maintenance at 50% methanol for the next 5 min at a flow rate of 0.4 mL/min [30].

### 2.6. Preparation of Starter Culture and Rice Bran Fermentation

Based on the above test results, a single LAB strain was selected as the rice bran fermentation starter and cultured in MRS broth for 24 h at 30 °C, harvested (9950× *g*, 15 min, 4 °C), washed twice with sterile distilled water, and suspended in sterile distilled water containing the same volume of culture.

Rice bran powder was obtained from Henanum Co., Ltd. (Yangpyeong, Korea). The rice bran slurry consisted of 20% (*w*/*v*) rice bran powder supplemented with 2.0% (*w*/*v*) glucose, 3.0% corn steep liquor, and 1.0–3.0% arginine in distilled water and was autoclaved (121 °C, 15 min) and immediately cooled. The prepared starter culture was inoculated (1.0%; equivalent to ~6 log CFU/mL), fermented for 48 h at 30 °C, and filtered through four layers of sterile thin cloth; this fermented rice bran filtrate was used to investigate the effect of pH and to monitor cell survival. The fermented rice bran was freeze-dried (SFDSM12, Samwon, Seoul, Korea), ground using a hand blender (BW-3000, Buwon, Daegu, Korea), and subjected to amino acid determination and sensory evaluation. In the sensory evaluation test, raw rice bran and freeze-dried non-fermented rice bran were also used as controls.

### 2.7. Characterization of Fermented Rice Bran

#### 2.7.1. pH and LAB Counts

The pH of the fermented rice bran was measured using a pH meter (Fisher Science Education, Hanover Park, IL, USA). The viable cell contents in fermented rice bran were determined by plating onto MRS agar and MRS + 2.0% CaCO_3_ agar [26].

#### 2.7.2. Amino Acid Contents

Distilled water was added to the prepared freeze-dried fermented rice bran, mixed well, ultrasonicated (PowerSonic 420, Hwasin Tech, Seoul, Korea), and stirred at room temperature for 1 h, and then the mixture was filtered using a 0.2 μm regenerated cellulose membrane filter (Sartorius, Göttingen, Germany). The arginine, citrulline, and ornithine contents in the filtrate were determined by HPLC (Section 2.3).

#### 2.7.3. Sensory Evaluation

The sensory evaluations were approved by the Institutional Review Board, Chosun University, Korea (IRB#2-1041055-AB-N-01). Nine trained students with previous experience of sensory evaluations of food, who had conducted more than 20 analyses per year at the Department of Food and Nutrition at Chosun University (Gwangju, Korea), participated in the evaluation of the fermented rice bran. Each sample (5 g) was served on a white plate, and the sample orders were randomized. The panelists rinsed their mouths with water before scoring the samples and waited for 1–2 min before evaluating subsequent samples. Sensory evaluation was performed by scoring the bitterness, saltiness, savory flavor, hay smell, mouthfeel texture, and overall acceptability using a 5-point scale, where 1 = very bad, 3 = moderate, and 5 = very good.

### 2.8. Statistical Analysis

Data are presented as the means and standard deviations (means ± SDs) of two or three independent experiments performed in duplicate. Statistical analysis was performed using Duncan’s multiple range test (DMRT) for one-way ANOVA and the independent-samples t test. The analysis was performed using SPSS version 26.0 for Windows (SPSS, Chicago, IL, USA), and statistical significance was accepted for *p*-values < 0.05.

## 3. Results and Discussion

### 3.1. Isolation and Identification of Ornithine-Producing LAB

Forty LAB strains were isolated from 38 kimchi samples collected from 29 regions of South Korea. Of these 40 isolates, 11 found to produce ornithine by TLC analysis (data not shown) were selected for further experimentation. All 11 isolates were Gram-positive and catalase-negative; one isolate was coccus-shaped, and other 10 were short-rod-shaped cells. When the 16S rRNA gene sequences (1373–1518 bp) of the 11 isolates were compared with those of LAB strains in GeneBank, one isolate (coccus-shaped) showed 99.38% homology with *Leuconostoc citreum* ATCC 49370^T^, whereas the others (short-rod shaped) showed 100% homology with *W. koreensis* JCM 11263^T^ (Supplementary Table S1). RAPD-PCR using strain- and species-specific primers has been shown to enable the identification of strains belonging to subspecies of the same species [13]. Thus, the genotypic differences among these 10 closely related strains were analyzed by RAPD-PCR. Of the 10 strains, five strains displaying slightly different RAPD patterns under different annealing temperatures (30–55 °C) were selected (Supplementary Figure S1) and investigated for ornithine production by HPLC. Two isolates, designated *Weissella koreensis* DB1 and HJ, produced significantly more ornithine than the others, consumed more arginine (Table 1), and were selected for further experimentation.

### 3.2. Safety Assessment

LAB are generally regarded as safe (GRAS), but it has been reported that some members of the genera *Lactobacillus*, *Leuconostoc*, *Pediococcus*, and *Bifidobacterium* can sometimes cause infections [31]. Because the health-promoting effects of LAB have led to their increased use in fermented foods, new LAB strains are continuously being sought for use as novel starter cultures or probiotics. However, new LAB strains require comprehensive testing to ensure they have no undesirable properties such as harmful biochemical effects, antibiotic resistance, or expressing virulence factors [10]. Thus, the efficacy of new LAB strains intended for use in starter cultures must be carefully assessed to determine their safety status. In this study, the new two LAB isolates, *W. koreensis* DB1 and HJ, were assessed for safety as follows.

#### 3.2.1. Undesirable Enzymatic Activities

The enzymatic activities of selected *W. koreensis* DB1 and HJ were examined using an API-ZYM kit (Table 2). *W. koreensis* DB1 and HJ did not show undesirable enzyme activities, especially β-glucuronidase or α-chymotrypsin activities, which have been reported to have negative effects in the colon [10]. β-Galactosidase hydrolyzes lactose to glucose and galactose, and *W. koreensis* DB1 showed β-galactosidase activity at 10 nmol. *W. koreensis* DB1 was determined to be lactose-negative in carbohydrate assimilation [32], which suggested *W. koreensis* DB1 has β-galactosidase but that its activity is too low to hydrolyze lactose completely. According to the manufacturer’s instructions, the hydrolysis of >20 nmol of substrate is deemed positive. Thus, the two strains exhibited negative reactions to all the enzymes according to API-ZYM analysis.

#### 3.2.2. Antibiotic Resistance and Hemolysis

*Weissella* species have not yet been granted Qualified Presumption of Safety (QPS) status by the European Commission’s European Food Safety Authority [33], and thus, a breakpoint has not been recommended for the *Weissella* species. *Weissella* species are closely related to *Leuconostoc* species and were considered *Leuconostoc* species until the genus *Weissella* was proposed by Collins et al. based on 16S rRNA phylogenetic analysis results [34]. Thus, the breakpoints for *Leuconostoc* species as stated by the EFSA and the MICs of other *Weissella koreensis* strains reported in the literature were used in this study. As shown in Table 3, the two LAB isolates had lower MICs than those highlighted for *Leuconostoc* by the EFSA. Furthermore, the MICs of the antibiotics tested were definitely lower than those reported for other *Weissella koreensis* strains [35,36]. Breakpoints of vancomycin for *Leuconostoc* species are not required by the technical guidelines of the EFSA [27], as *Leuconostoc* species are intrinsically vancomycin-resistant, unlike other general antibiotic resistance mechanisms [37]. The results in Table 3 show that *W. koreensis* DB1 and HJ are susceptible to all the antibiotics except vancomycin.

*W. koreensis* DB1 and HJ did not show α- or β-hemolytic activities on horse blood agar in the hemolysis assays. However, Jeong and Lee have reported that two *Weissella* isolates from kimchi exhibited α-hemolytic activity but no β-hemolysis [28].

#### 3.2.3. Biogenic Amine Production

The enzymatic activities of *W. koreensis* DB1 and HJ can produce ornithine from arginine via the ADI pathway (Table 1). In particular, the biogenic amines (BAs) putrescine and agmatine can be produced by the microbial decarboxylation of ornithine and arginine [38]. We investigated whether the two isolates produce BAs via amino acid decarboxylation or via a combination of decarboxylation and the ADI pathway. The consumption of food containing high BAs is a risk for human health, as BAs have toxic effects [38]; it has been proposed that LAB starters lacking the ability to accumulate BA are needed to develop high-quality foods [15]. It has also been reported that some *Weissella* strains can produce BAs such as putrescine, tyramine, and histamine [28,39]. Thus, the two LAB were cultivated in MRS + 1.0% arginine for 48 h, and then, the cultures were analyzed for BAs. As shown in Supplementary Figure S2, neither strain produced agmatine nor putrescine.

We believe it is reasonable to conclude that the consumption of *W. koreensis* DB1 and HJ does not represent a health risk based on the results of our safety assessment.

### 3.3. Effects of Culture Conditions on LAB Growth

#### 3.3.1. Effect of Temperature

**Figure 1:**
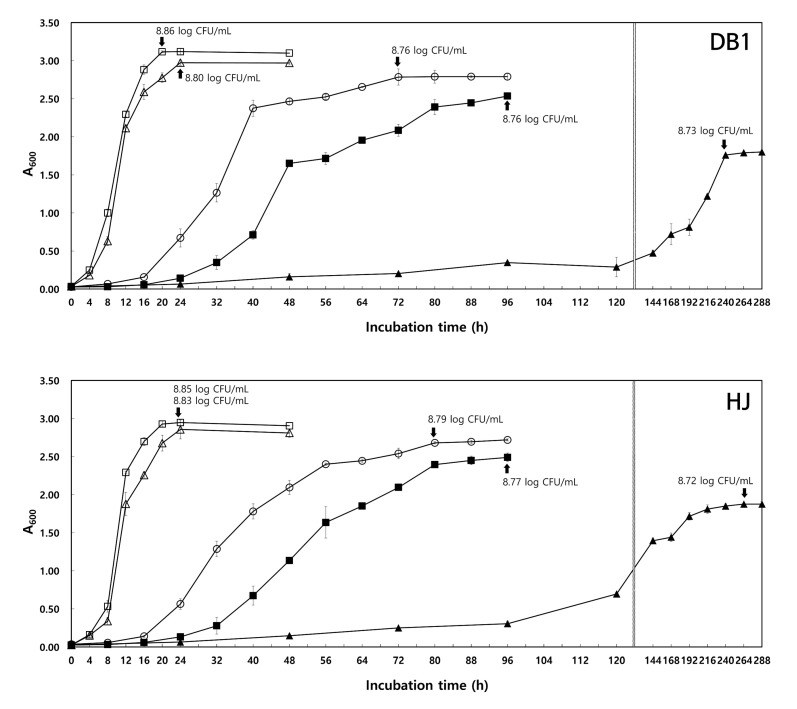
Growth of LAB at different temperatures. *W. koreensis* DB1 and HJ were incubated in MRS broth at 5 °C (▲), 10 °C (■),15 °C (○), 25 °C (△), or 30 °C (□) for 24–288 h.

The highest cell growth in MRS broth was obtained at 30 °C, and this decreased progressively with temperature. The optimum growth temperature for *W. koreensis* DB1 and HJ was 30 °C, and they reached the stationary phase at 20–24 h, with maximum cell viabilities of 8.85–8.86 log CFU/mL in MRS broth. *W. koreensis* DB1 and HJ reached the stationary phase at 240 and 264 h, respectively, when cultivated at 5 °C and showed 8.72–8.73 log CFU/mL of viable cells (Figure 1). These results are consistent with a report that described that *W. koreensis*, a psychrophilic bacterium, grows well under mesophilic and psychrophilic conditions [15].

#### 3.3.2. Effect of pH

**Figure 2:**
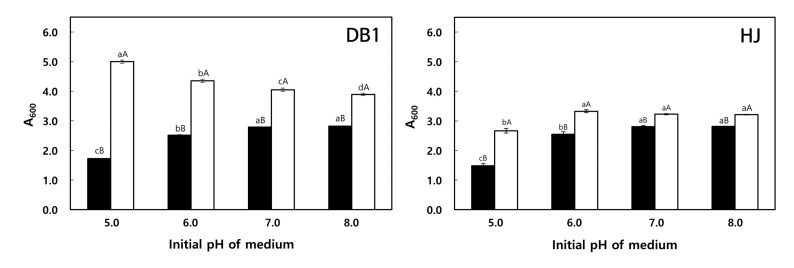
Effect of initial pH on LAB growth. Initial pH values of MRS (■) and MRS + 1.0% arginine (□) were adjusted to 5.0–8.0, and then, *W. koreensis* DB1 or HJ were cultivated in these media for 24 h at 30 °C. Different lowercase letters indicate significant differences (*p* < 0.05) between cultures with different initial pH values as determined by Duncan’s multiple range test. Different uppercase letters indicate significant differences (*p* < 0.05) between cultures with or without arginine supplementation as determined by the independent-samples t test.

*W. koreensis* DB1 and HJ were cultivated at 30 °C for 24 h in MRS or MRS + 1.0% arginine medium adjusted to initial pH values of 5.0, 6.0, 7.0, or 8.0 (Figure 2). The cell growth of the two isolates was enhanced with arginine supplementation, but this growth enhancement was greater for *W. koreensis* DB1. It has been reported that supplementation with arginine enhances bacterial growth, as bacteria use arginine as an energy source via the ADI system [40]. *W. koreensis* DB1 showed the greatest cell growth at pH 5.0 in MRS + 1.0% arginine and at pH 7.0–8.0 in MRS. The reason for these different pH optima is presumed to be due to different amounts of NH_3_ being produced in MRS and MRS + 1.0% arginine cultures. That is, the NH_3_ produced via the ADI pathway in MRS + 1.0% arginine neutralizes the initial pH of 5.0 and the organic acids produced by glucose metabolism. The neutralizing effect of the NH_3_ produced by *W. koreensis* DB1 in MRS + arginine might also aid bacterial growth in culture. On the other hand, *W. koreensis* DB1 cultured in MRS produced organic acids but not NH_3_, and thus, an initial pH of 5.0 in MRS inhibited the growth.

On the other hand, *W. koreensis* HJ showed the lowest cell growth at pH 5.0 and highest cell growth at pH 7.0–8.0 in MRS and at pH 6.0–8.0 in MRS + 1.0% arginine. *W. koreensis* HJ can also degrade arginine via the ADI pathway and produce NH_3_, but it appears that the arginine catabolic ability of *W. koreensis* HJ was not enough to neutralize the initial pH of 5.0 and the organic acids produced when it was cultured in MRS + arginine. These results imply that the ornithine-producing capacity of *W. koreensis* DB1 is greater than that of *W. koreensis* HJ, which is in line with the results shown in Table 1.

Based on the results shown in Figure 2, we concluded that the optimum initial pH for *W. koreensis* DB1 in MRS + 1.0% arginine was 5.0, whereas that for *W. koreensis* HJ was 8.0, because *W. koreensis* HJ had a slightly higher viable cell count at pH 8.0 (7.93 log CFU/mL at pH 6.0 and 8.36 log CFU/mL at pH 8.0 in MRS + 1.0% arginine).

#### 3.3.3. Effect of Arginine Concentration

**Figure 3:**
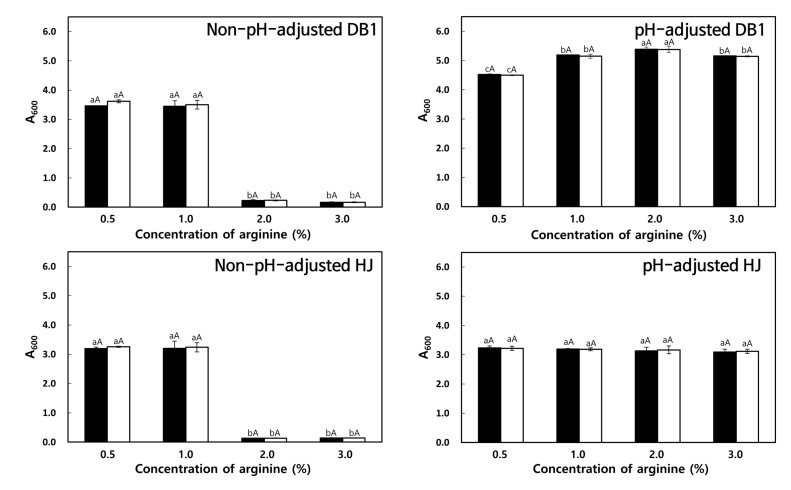
Effect of arginine concentration in MRS on LAB growth. *W. koreensis* DB1 and *W. koreensis* HJ were cultivated in non-pH-adjusted (left side) or pH-adjusted (right side; pH 5.0 for *W. koreensis* DB1 and pH 8.0 for *W. koreensis* HJ) MRS + 1.0% arginine for 24 h (■) or 48 h (□) at 30 °C. Different lowercase letters indicate significant differences (*p* < 0.05) between cultures with different arginine concentrations as determined by Duncan’s multiple range test. Different uppercase letters indicate significant differences (*p* < 0.05) between cultivation times as determined by the independent-samples *t* test.

When *W. koreensis* DB1 and HJ were cultivated in MRS supplemented with 0.5–3.0% arginine without pH adjustment, the isolate growth was obviously low (Figure 3—left side). In particular, in the presence of 2.0–3.0% arginine, little growth was observed for 24–48 h. However, when the pH values were adjusted to pH 5.0 for *W. koreensis* DB1 and pH 8.0 for *W. koreensis* HJ, cell growth was significantly improved, especially in MRS supplemented with 1.0–3.0% arginine (Figure 3—right side).

**Figure 4:**
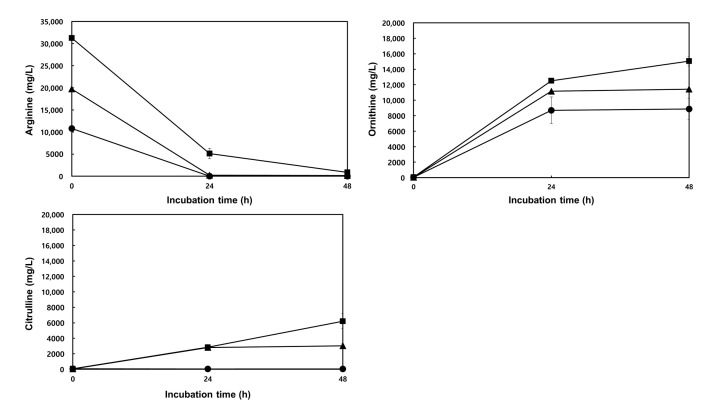
Ornithine and citrulline production by *W. koreensis* DB1. *W. koreensis* DB1 was cultivated in MRS broth supplemented with 1.0% (●), 2.0% (▲), or 3.0% (■) arginine (initial pH, 5.0) at 30 °C for 24–48 h. Levels of arginine, citrulline, and ornithine in cultures were determined by HPLC as described in Materials and Methods.

Ornithine production by *W. koreensis* DB1 in MRS supplemented with 1.0–3.0% arginine for 24–48 h (adjusted to an initial pH of 5.0) at 30 °C was quantified by HPLC (Figure 4). The highest ornithine (15,059.65 mg/L) and citrulline (6206.03 mg/L) production with 97.17% arginine conversion was obtained for 3.0% arginine supplementation and cultivation for 48 h, whereas the lowest ornithine (8697.99 mg/L) and citrulline (38.23 mg/L) production was obtained with a 99.83% conversion of arginine for 1.0% arginine supplementation and cultivation for 24 h. Arginine is degraded by the ADI pathway into citrulline, ornithine, CO_2_, and NH_3_ [15]. The supplemented arginine (1.0–3.0%) levels rapidly decreased, and arginine was mainly converted to ornithine, for 24 h. Citrulline was also produced as a metabolic intermediate and accumulated when arginine was supplemented at levels >2.0%.

### 3.4. Characteristics of the Fermented Rice Bran

#### 3.4.1. LAB Growth and Ornithine Production

High cell growth is one of the important factors for producing ornithine. *W. koreensis* DB1 was cultured in 20% rice bran +1.0% arginine supplemented with 1.0–3.0% glucose and 1.0–5.0% corn steep liquor at 30 °C for 48 h, and then, cell growth was determined. As shown in Supplementary Table S2, the highest cell growth was obtained from the addition of 2.0% glucose and 3.0% corn steep liquor. Based on the results of Supplementary Table S2, we prepared rice bran slurries composed of 20% rice bran +2.0% glucose + 3.0% corn steep liquor supplemented with 1.0% (A), 2.0% (B), or 3.0% (C) arginine in distilled water. The slurries were autoclaved (121 °C, 15 min), and then, *W. koreensis* DB1 was inoculated (at ~6 log CFU/mL). When the initial pH of the slurries was adjusted to 5.0 (based on the results shown in Figure 2), no *W. koreensis* DB1 growth was observed, which indicated that an initial pH of 5.0 was unsuitable for *W. koreensis* DB1 cultivation. This is presumably due to differences between the nutritional compositions of MRS and rice bran slurries. Actually, the optimum pH for the growth of most LAB is 6.0–7.0 [41], and thus, the initial pH of the commercialized MRS (Difco, Sparks, MD, USA) is pH 6.5 ± 0.2. The composition of MRS is optimized for LAB growth. In MRS + arginine (even though its initial pH was adjusted to pH 5.0), rapid cell growth occurred, and consequently, the NH_3_ produced neutralized the initial acidity and enhanced cell growth (Figure 2). However, the rice bran slurry used in the present study was not a growth medium optimized for LAB, and this probably inhibited *W. koreensis* DB1 growth with an initial pH of 5.0.

When the initial pH of the rice bran slurry was adjusted to pH 6.0 and the slurry was then fermented at 30 °C for 48 h, *W. koreensis* DB1 grew well, and this resulted in almost the same viable cell count as cultivation in MRS + 1.0% arginine (pH 6.0, 8.58 log CFU/mL). *W. koreensis* DB1 growth in rice bran slurry reached 8.61–8.72 log CFU/mL after 48 h of fermentation, and the resultant pH values were 5.54 for A, 6.37 for B, and 7.16 for C (Table 4). Thus, the final pH values of the fermented rice bran increased with the initial arginine concentration, which suggests that higher initial arginine concentrations resulted in greater arginine catabolism by *W. koreensis* DB1 (Table 4). Lactic and acetic acids were detected at 16,253.65 and 1723.24 mg/kg, respectively, at 0 h due to the addition of 3.0% corn steep liquor, which contains both. After 48 h of fermentation, the highest organic acid concentration (lactic and acetic acids; 60,952.93 mg/kg) was detected for A and the lowest organic acid concentration (50,068.36 mg/kg) was detected for C, which was consistent with the pH results.

In fact, 99.39–99.61% of the arginine supplemented at 1.0–3.0% was consumed by the production of ornithine, citrulline, and other ADI metabolites. The highest ornithine yield (43,074.13 mg/kg) was obtained for rice bran fermentation supplemented with 3.0% arginine, which also resulted in a citrulline yield of 27,336.37 mg/kg. In our previous preliminary study, rice bran was fermented under non-optimized fermentation conditions. In detail, rice bran slurries were prepared with 20% rice bran +1.0% glucose +1.0% arginine without pH adjustment in distilled water. The pH of the prepared rice bran was 6.84 due to arginine addition. *W. koreensis* DB1 was inoculated (at 6 log CFU/mL) and used for fermentation for 48 h at 30 °C. After fermentation, the pH of the fermented rice bran was pH 6.61, and viable cells were detected at 8.15 log CFU/mL. Thereafter, the fermented rice bran was hot-air dried at 55 °C for 12 h, and then, the ornithine and citrulline contents were determined at 8446.78 and 3199.46 mg/kg, respectively [25]. However, the amount of ornithine produced was not enough to satisfy daily requirements for functional food; thus, the enhancement of ornithine in fermented rice bran was needed. Based on the results of our previous study and the results presented in this study, glucose, corn steep liquor, and arginine were added in rice bran fermentation. The addition of 2.0% glucose, 3.0% corn steep liquor, and 3.0% arginine in 20% rice bran slurry (the initial pH was adjusted to pH 6.0) clearly increased *W. koreensis* DB1 growth and ornithine production. The differences between the previous study and this study in terms of rice bran fermentation conditions were pH adjustment (to pH 6.0) and the rice bran slurry composition, namely, with respect to glucose, corn steep liquor, and arginine. After changing the fermentation conditions, the ornithine and citrulline contents were significantly greater than those observed during the previous study. l-ornithine has a variety of beneficial effects, such as stress reduction, physical fatigue reduction, anti-obesity effects, and others [15]. When used as a dietary supplement, l-citrulline appears to be a powerful pharmaconutrient that reduces hypertension, arterial stiffness, and lipid oxidation, and increases muscle mass, muscle performance, and fat-free mass [42]. Citrulline is almost absent in foods. Actually, only watermelons contain significant amounts of citrulline [42]. l-citrulline has been reported to provide an efficient intervention for increasing l-arginine bioavailability [43]. We found that *W. koreensis* DB1-fermented rice bran resulted in the production of high levels of ornithine (43,074.13 mg/L) and citrulline (27,336.37 mg/L).

#### 3.4.2. Sensory Evaluation

On performing a sensory evaluation as shown in Table 5, the fermented rice bran (FRB) showed a superior taste and flavor along with a soft mouthfeel. The FRB clearly received higher scores for overall preference than the others. The FRB presented its own characteristic balanced combination of savory flavor and moderately salty taste along with a soft mouthfeel texture. The savory flavor may have stemmed from the fermentative characteristics of *W. koreensis* DB1 and the moderately salty taste resulting from the neutralization of lactic acid by the ammonia produced by *W. koreensis* DB1.

Generally, rice bran addition in food products has an unacceptable effect due to its typical smell and texture [44]. However, our results indicated that the sensory quality of rice bran was substantially improved via *W. koreensis* DB1 fermentation. Raw rice bran (RRB) smells of hay and has a coarse mouthfeel and bland taste, and freeze-dried rice bran slurry without fermentation (SRB) is bitter due to the presence of arginine [45]. However, the bitterness of SRB and the hay-like smell and coarse texture of RRB were clearly removed by fermentation.

## 4. Conclusions

Rice bran is a by-product of rice polishing and is a rich source of dietary fiber (11.77–12.68%) [46]. The consumption of dietary fiber has beneficial effects on human health, as it ameliorates gastrointestinal concerns, improves lipid metabolism, and reduces cholesterol levels [46,47]. In addition, rice bran has prebiotic properties [47,48]. Consumers are ever more interested in the health-promoting attributes of food and their organoleptic properties. In this study, high-ornithine-producing *W. koreensis* DB1 isolated from kimchi was used as a starter culture for rice bran fermentation. Our results showed *W. koreensis* DB1 did not exhibit any harmful characteristics, and we concluded that *W. koreensis* DB1 is suitable as a safe starter culture for the development of high-quality foods. Compared to the results of the previous study, this study reports significant increases in ornithine and citrulline contents under optimal rice bran fermentation conditions for *W. koreensis* DB1. Ten grams of *W. koreensis* DB1-fermented rice bran was found to contain sufficient ornithine (43,074.13 mg/kg) to satisfy daily requirements (400–1000 mg). It also contained around 27,336.37 mg/kg of citrulline and viable LAB *W. koreensis* DB1 (8.65 log CFU/mL), and was rich in dietary fiber. Moreover, fermented rice bran showed substantially improved organoleptic quality. These results indicate that the fermented rice bran produced during this study is a promising functional food candidate with excellent composition, functionality, and organoleptic quality. The health beneficial effects of the fermented rice bran produced will be confirmed by further pre-clinical trials in animals and clinical trials in human subjects.

## Figures and Tables

**Table 1:** Ornithine production from arginine by lactic acid bacteria (LAB) isolates.

Amino Acid (mg/L)	*Weissella koreensis*				
	GL	DB1	CM	CGM1	HJ
Arginine	93.35 ± 0.92 ^b^	55.46 ± 2.07 ^c^	248.98 ± 26.13 ^a^	102.03 ± 1.02 ^b^	60.69 ± 0.71 ^c^
Ornithine	4963.02 ± 23.18 ^c^	6581.64 ± 115.09 ^a^	3904.86 ± 101.73 ^d^	4922.22 ± 86.31 ^c^	5975.50 ± 126.94 ^b^

LAB isolates were incubated in de Man, Rogosa, and Sharpe (MRS) + 1.0% arginine at 30 °C for 48 h, and then, arginine and ornithine contents were analyzed by HPLC. Values are the means ± SDs of two independent cultivations. Means with different letters (a–d) in the same row were significantly different (*p* < 0.05) as determined by Duncan’s Multiple Range Test.

**Table 2:** API-ZYM analysis of LAB enzyme activities.

Enzyme (nmol)	Activity of *W. koreensis*	
	DB1	HJ
Alkaline phosphate	0	0
Esterase (C4)	0	0
Esterase lipase (C8)	0	0
Lipase (C14)	0	0
Leucine arylamidase	0	10
Valine arylamidase	0	0
Cystine arylamidase	0	0
Trypsin	0	0
α-Chymotrypsin	0	0
Acid phosphatase	0	5
Naphthol-AS-BI-phosphohydrolase	5	5
A-Galactosidase	0	0
β-Galactosidase	10	0
β-Glucuronidase	0	0
α-Glucosidase	0	0
β-Glucosidase	0	0
N-Acetyl-β-glucosaminidase	0	0
α-Mannosidase	0	0
α-Fucosidase	0	0

Enzymatic activities are presented as nanomoles of hydrolyzed substrate after 4 h of incubation at 37 °C; 0 = no activity, 5 = 5 nmol, and 10 = 10 nmol. According to the manufacturer’s instructions, ≥20 nmol of substrate hydrolyzed was defined as a positive reaction and <20 nmol of substrate hydrolyzed as a negative reaction.

**Table 3:** Minimum inhibitory concentrations (MICs) of antibiotics for LAB.

Antibiotics (μg/mL)	Breakpoints for *Leuconostocs* ^1^	MICs for Other *Weissella koreensis* ^2^	*W. koreensis*	
			DB1	HJ
Ampicillin	2	1~>10	0.5	1
Vancomycin	N.R ^3^	1024	512	512
Gentamycin	16	>10–32	2	4
Kanamycin	16	30–512	8	8
Streptomycin	64	128	8	4
Erythromycin	1	1~>15	0.06	0.25
Tetracycline	8	1	0.5	0.5
Chloramphenicol	4	4~>30	4	4

^1^ Breakpoints quoted are in accord with European Food Safety Authority (EFSA) guidelines (2012) [27]. ^2^ MICs for other *Weissella koreensis* strains were determined by other investigators [35,36]. ^3^ N.R: Not required.

**Table 4:** pH, viable cell, and amino acid content changes in fermented rice bran.

Fermented Rice Bran	Characteristics	Fermentation time	
		0 h	48 h
A	pH	6.00 ± 0.01 ^a^	5.54 ± 0.20 ^b^
	Viable cells (log CFU/mL)	6.07 ± 0.12 ^b^	8.61 ± 0.09 ^a^
	Arginine (mg/kg)	27,986.04 ± 279.01 ^a^	169.33 ± 82.95 ^b^
	Citrulline (mg/kg)	74.10 ± 9.10 ^b^	5444.79 ± 364.49 ^a^
	Ornithine (mg/kg)	17.10 ± 2.10 ^b^	19,972.33 ± 1255.01 ^a^
	Lactic acid (mg/kg)	16,253.65 ± 477.16 ^b^	58,743.83 ± 673.83 ^a^
	Acetic acid (mg/kg)	1723.24 ± 50.59 ^b^	2209.00 ± 25.34 ^a^
B	pH	6.00 ± 0.01 ^b^	6.37 ± 0.02 ^a^
	Viable cells (log CFU/mL)	6.06 ± 0.06 ^b^	8.72 ± 0.15 ^a^
	Arginine (mg/kg)	64,950.08 ± 425.02 ^a^	256.26 ± 25.79 ^b^
	Citrulline (mg/kg)	127.23 ± 6.90 ^b^	16,113.25 ± 2321.96 ^a^
	Ornithine (mg/kg)	68.02 ± 7.03 ^b^	32,747.80 ± 1421.15 ^a^
	Lactic acid (mg/kg)	16,253.65 ± 477.16 ^b^	52,493.50 ± 1100.15 ^a^
	Acetic acid (mg/kg)	1723.24 ± 50.59 ^b^	3736.31 ± 1113.09 ^a^
C	pH	6.00 ± 0.00 ^b^	7.16 ± 0.10 ^a^
	Viable cells (log CFU/mL)	6.07 ± 0.09 ^b^	8.65 ± 0.10 ^a^
	Arginine (mg/kg)	91,813.04 ± 608.01 ^a^	464.14 ± 33.94 ^b^
	Citrulline (mg/kg)	188.07 ± 8.05 ^b^	27,336.37 ± 4870.86 ^a^
	Ornithine (mg/kg)	442.10 ± 55.06 ^b^	43,074.13 ± 2457.05 ^a^
	Lactic acid (mg/kg)	16,253.65 ± 477.16 ^b^	45,380.99 ± 1258.69 ^a^
	Acetic acid (mg/kg)	1723.24 ± 50.59 ^b^	4687.37 ± 183.08 ^a^

Rice bran slurry was composed of 20% rice bran powder + 2.0% glucose + 3.0% corn steep liquor supplemented with 1.0% (A), 2.0% (B), or 3.0% (C) arginine in distilled water. The slurry was autoclaved (121 °C, 15 min), and *W. koreensis* DB1 was inoculated (~6 log CFU/mL) and used for fermentation at 30 °C for 48 h. pH and viable cell counts were then determined. The fermented rice bran slurry (0 or 48 h) was freeze-dried and then ground, and amino acid contents were analyzed by HPLC. Values are the means ± SDs of three independent cultivations. Means with different letters (a–b) in the same row were significantly different (*p* < 0.05) as determined by the Independent Samples *T* Test.

**Table 5:** Sensory evaluation of fermented rice bran.

Items	RRB	SRB	FRB
Bitterness	3.8 ± 0.8 ^a^	1.8 ± 0.4 ^b^	3.5 ± 0.8 ^a^
Saltiness	1.9 ± 0.7 ^b^	3.5 ± 0.8 ^a^	3.8 ± 0.4 ^a^
Savory flavor	2.3 ± 0.9 ^c^	3.3 ± 0.6 ^b^	4.5 ± 0.5 ^a^
Hay smell	1.5 ± 0.5 ^c^	3.6 ± 0.5 ^b^	4.2 ± 0.6 ^a^
Mouthfeel texture	1.7 ± 0.5 ^c^	3.7 ± 0.8 ^b^	4.3 ± 0.5 ^a^
Overall acceptability	1.7 ± 0.5 ^c^	2.8 ± 0.8 ^b^	4.4 ± 0.5 ^a^

Rice bran slurry was composed of 20% raw rice bran powder (RRB) supplemented with 2.0% glucose, 3.0% corn steep liquor, and 3.0% arginine in distilled water. The slurry was autoclaved, and then, *W. koreensis* DB1 was inoculated (~6 log CFU/mL); the slurry was then fermented at 30 °C for 0 h (SRB) or for 48 h (FRB), freeze-dried, and ground. Sensory evaluation was carried out using the prepared RRB, SRB, and FRB samples and rated using a 5-point scale, in which 1, 3, and 5 corresponded to very bad, moderate, and very good, respectively. Values are the means ± SDs of duplicate determinations. Means with different letters (a–c) in the same row were significant differences (*p* < 0.05), as determined by Duncan’s Multiple Range Test.

## Supplementary Materials

The following are available online at https://www.mdpi.com/2304-8158/9/11/1545/s1. Figure S1: RAPD patterns of LAB. Bands distinctly distinct among LAB strains are marked with red boxes. LAB strains displaying different RAPD patterns after annealing at different temperatures are indicated as ①–⑤, Figure S2: HPLC chromatogram of biogenic amines. Biogenic amine standards: PUT, putrescine; HIS, histamine; AGM, agmatine; and IS, 1,7-diaminoheptane (the internal standard) (A). Culture supernatant of *W. koreensis* DB1 (B). Culture supernatant of *W. koreensis* HJ (C). Figure S3: TLC analysis of LAB cultures. *W. koreensis* DB1 and *W. koreensis* HJ were incubated in MRS broth supplemented with 0.5–3.0% arginine for 24 h (A) or 48 h (B). Cultures were then analyzed by TLC as described in Materials and Methods. 1: arginine standard, 2: ornithine standard, 3–6: initial pHs of the cultures were adjusted to pH 5.0 for DB1 and to pH 8.0 for HJ, and 7–10: pHs of cultures were not adjusted (controls). Table S1: Similarities of the 16S rRNA gene sequences of LAB isolates with those in the GenBank database. Table S2: Viable cell determination according to addition of corn steep liquor and glucose in rice bran fermentation.
