# Supplementary material for: Characterization of High-Ornithine-Producing Weissella koreensis DB1 Isolated from Kimchi and Its Application in Rice Bran Fermentation as a Starter Culture
Source: Foods. 2020 Oct 26;9(11):1545. doi: 10.3390/foods9111545 (PMC7693252; doi:10.3390/foods9111545)
Supplement: Supplementary file 1 [file foods-09-01545-s001.pdf]

Supplementary data

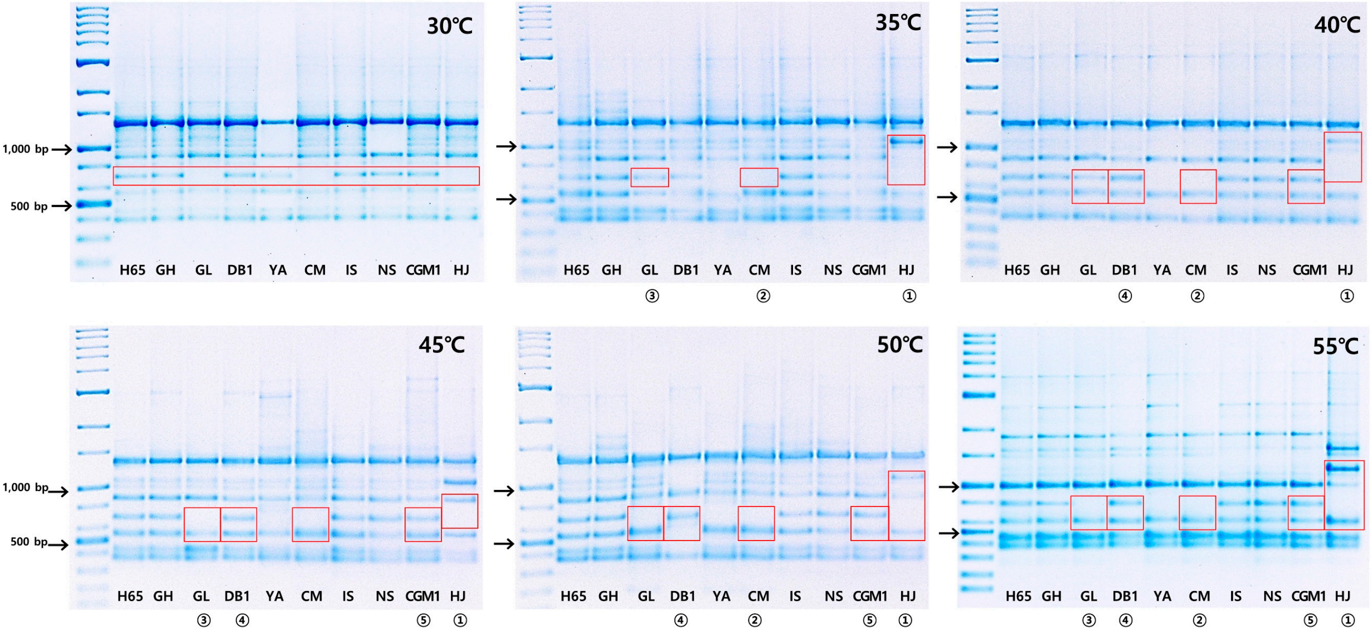

Supplementary Figure 1. RAPD-patterns of LAB. Bands distinctly distinct among LAB strains are marked with red boxes. LAB strains displaying different RAPD patterns after annealing at different temperatures are indicated as ①~⑤.

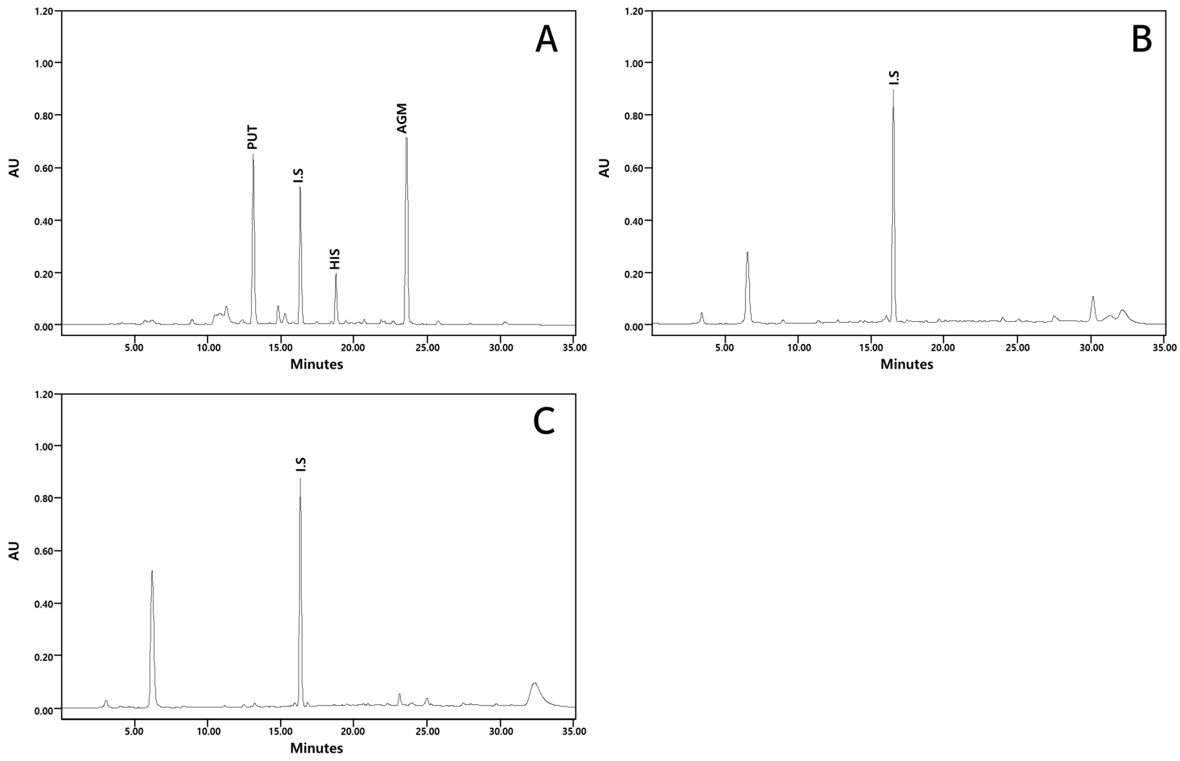

Supplementary Figure 2. HPLC chromatogram of biogenic amines. Biogenic amine standards, PUT; putrescine, HIS; histamine, AGM; agmatine, and IS; 1,7-diaminoheptane (the internal standard) (A). Culture supernatant of *W. koreensis* DB1 (B). Culture supernatant of *W. koreensis* HJ (C).

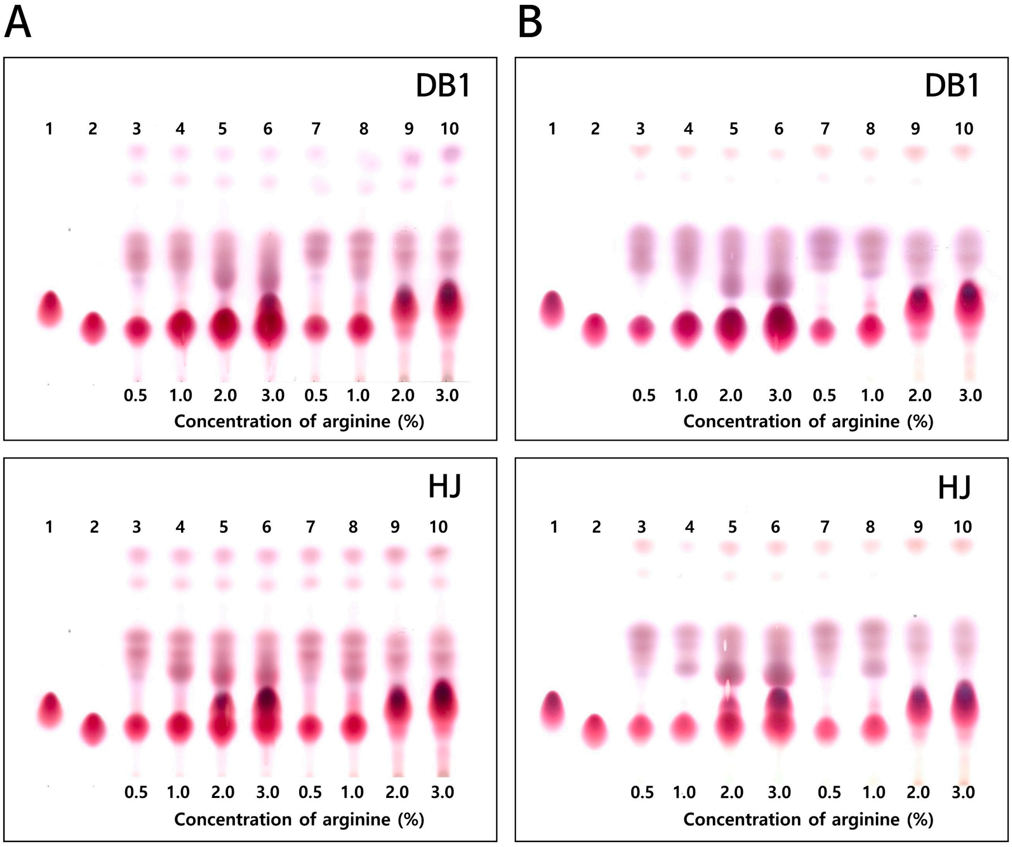

Supplementary Figure 3. TLC analysis of LAB cultures. *W. koreensis* DB1 and *W. koreensis* HJ were incubated in MRS broth supplemented with 0.5~3.0% arginine for 24 h (A) or 48 h (B). Cultures were then analyzed by TLC as described in Materials and Methods. 1: arginine standard, 2: ornithine standard, 3~6: initial pHs of the cultures were adjusted to pH 5.0 for DB1 and to pH 8.0 for HJ, and 7~10: pHs of cultures were not adjusted (controls).

Supplementary Table 1. Similarities of the 16S rRNA gene sequences of LAB isolates with those in the GenBank database.

No.	Isolated strain	Length of 16S rRNA gene (bp)	Identified LAB	Similarity (%)	E value	GenBank Accession No.
1	H65	1,442	*Weissella koreensis* JCM 11263^T	100.00	0.0	MT856440
2	GH	1,504	*Weissella koreensis* JCM 11263^T	100.00	0.0	MT856470
3	GL	1,511	*Weissella koreensis* JCM 11263^T	100.00	0.0	MT856476
4	DB1	1,460	*Weissella koreensis* JCM 11263^T	100.00	0.0	MH450055
5	YA	1,373	*Weissella koreensis* JCM 11263^T	100.00	0.0	MT856478
6	CM	1,518	*Weissella koreensis* JCM 11263^T	100.00	0.0	MT856647
7	IS7	1,515	*Weissella koreensis* JCM 11263^T	100.00	0.0	MT856648
8	NS1	1,512	*Weissella koreensis* JCM 11263^T	100.00	0.0	KU365163
9	CGM1	1,453	*Weissella koreensis* JCM 11263^T	100.00	0.0	MT856649
10	HJ	1,518	*Weissella koreensis* JCM 11263^T	100.00	0.0	MH450054
11	EB4	1,442	*Leuconostoc citreum* ATCC 49370^T	99.38	0.0	MT856651

Supplementary Table 2. Viable cell determination according to addition of corn steep liquor and glucose in rice-bran fermentation.

Basic Rice-bran slurry	Corn steep liquor concentration	Glucose concentration	Viable cells (log CFU/mL)
20% Rice-bran + 1.0% arginine	1.0%	1.0%	8.12 ± 0.15 [bc]
		2.0%	8.09 ± 0.18 [cd]
		3.0%	8.02 ± 0.07 [cd]
	3.0%	1.0%	8.16 ± 0.24 [bc]
		2.0%	8.60 ± 0.07 [a]
		3.0%	8.33 ± 0.05 [b]
	5.0%	1.0%	7.89 ± 0.16 [d]

Rice-bran slurry was composed of 20% rice-bran + 1% arginine supplemented with glucose 1.0~3.0% and corn steep liquor 1.0~5.0% in distilled water. The slurry was autoclaved (121 °C, 15 min) and *W. koreensis* DB1 was inoculated (~6 log CFU/ml) and fermented at 30 °C for 48 h. Thereafter viable cell counts were determined. Values are the means ± SDs of three independent cultivations. Means with different letters in the same column were significantly different ($p<0.05$) as determined by Duncan's Multiple Range Test.
